# Effects of Eight Weeks of Unilateral Complex Training on Musculoskeletal Function, Inter-Limb Strength Asymmetry, and Athletic Performance in Competitive Basketball Players

**DOI:** 10.3390/life16091492

**Published:** 2026-09-06

**Authors:** Gizem Akarsu Taşman, Erkan Güven, Nasuh Evrim Acar, Bilal Gök, Zarife Pancar

**Affiliations:** 1 Faculty of Sports Sciences, Mersin University, Mersin 33110, Türkiye; gizemakarsu88@gmail.com (G.A.T.); erkanguven@mersin.edu.tr (E.G.); nasuhacar@mersin.edu.tr (N.E.A.); 2 Exercise and Sports Sciences Application and Research Center, Mersin University, Mersin 33110, Türkiye; 3 Faculty of Sports Sciences, Istanbul Gelişim University, Istanbul 34310, Türkiye; bigok@gelisim.edu.tr; 4 Department of Physical Education and Sports, Faculty of Sports Sciences, Gaziantep University, Gaziantep 27350, Türkiye

**Keywords:** basketball, unilateral complex training, isokinetic strength, bilateral strength asymmetry, H/Q ratio, physical performance

## Abstract

Background: Unilateral complex training has been proposed as an effective strategy to improve lower-limb neuromuscular function; however, its effects on isokinetic strength, inter-limb strength asymmetry, and athletic performance in competitive basketball players remain insufficiently investigated. This study examined the effects of an eight-week unilateral complex training program on lower-limb isokinetic strength, strength asymmetry, hamstring-to-quadriceps (H/Q) ratio, and athletic performance in young male basketball players. Methods: Twenty-nine competitive male basketball players (age: 19.14 ± 0.99 years) competing in the Turkish Basketball Youth League and U18 League were randomly assigned to an experimental group (*n* = 15) or a control group (*n* = 14). The experimental group performed unilateral complex training twice weekly for eight weeks in addition to regular basketball practice, whereas the control group continued routine basketball training only. Before and after the intervention, lower-limb isokinetic strength (60°·s^−1^), bilateral strength asymmetry, H/Q ratio, countermovement jump (CMJ), Abalakov jump, 20-m sprint performance, and force-platform-derived jump variables were assessed. Data were analyzed using two-way mixed-design analysis of variance. Results: Significant Group × Time interactions were observed for right and left knee flexor strength and left knee extensor strength (all *p* < 0.05). Bilateral quadriceps and hamstring strength asymmetries were significantly reduced by approximately 47% and 63%, respectively, accompanied by an improvement in the left H/Q ratio (*p* < 0.05). The intervention also produced significant improvements in CMJ height, Abalakov jump performance, flight time, time to takeoff, and 20-m sprint performance (all *p* < 0.05), whereas no significant interaction was found for peak force (*p* > 0.05). Conclusions: Adding an eight-week unilateral complex training program to regular basketball training was associated with improvements in selected lower-limb isokinetic strength and athletic performance outcomes, reductions in measured inter-limb strength asymmetry indices, and a side-specific improvement in the left H/Q ratio in competitive young male basketball players. However, because the control group did not receive an additional time- and volume-matched training stimulus, these findings should be interpreted as the effects of adding the overall training program rather than as evidence of the specific effects of its unilateral or complex components.

## 1. Introduction

Basketball is an intermittent, high-intensity team sport characterized by frequent transitions between offensive and defensive play, requiring athletes to repeatedly perform explosive actions such as sprinting, jumping, rapid changes of direction, and accelerations throughout competition [1,2]. During a single match, players typically execute approximately 40–60 maximal jumps, 50–60 high-intensity change-of-direction movements and cover total distances ranging from 4300 to 7500 m [3]. These repeated high-intensity actions impose substantial neuromuscular and metabolic demands, making lower-limb strength, power, and fatigue resistance critical determinants of basketball performance [4,5,6]. Consequently, optimizing physical conditioning has become a primary objective for strength and conditioning coaches seeking to maximize athletic performance while simultaneously reducing injury risk [7,8]. Although numerous resistance- and power-training methods have been proposed to enhance these physical qualities, the most effective strategy for simultaneously improving performance and lower-limb neuromuscular function remains unclear.

Resistance and plyometric training are among the most commonly used training methods for improving lower-limb strength, explosive power, and athletic performance in basketball players [9]. However, implementing these two training modalities in separate sessions may complicate training periodization and time management, particularly during congested training and competitive schedules [10]. Consequently, complex training, which combines a high-intensity resistance exercise with a biomechanically similar plyometric exercise within the same training session, has gained considerable attention as a time-efficient strategy for enhancing athletic performance [11,12]. Complex training may acutely enhance subsequent explosive performance through post-activation performance enhancement (PAPE), which refers to a transient improvement in performance following a conditioning activity [12]. However, PAPE represents an acute response and should be distinguished from the chronic adaptations resulting from repeated complex training. Over longer training periods, improvements may instead reflect cumulative neuromuscular adaptations to repeated resistance and plyometric loading rather than PAPE itself.

In recent years, increasing attention has been directed toward unilateral, rather than bilateral, complex training. Compared with bilateral exercises, unilateral training has been reported to elicit greater neuromuscular activation, increase the recruitment of fast-twitch motor units, and improve force-producing capacity [13]. Furthermore, unilateral complex training has been shown to enhance sprint, jump, and other athletic performance outcomes across various sports [14,15,16]. In addition to improving performance, unilateral loading may preferentially stimulate the weaker limb, thereby contributing to reductions in inter-limb strength asymmetry [13]. Inter-limb asymmetry has been associated with differences in athletic performance, including sprinting, jumping, and change-of-direction ability; however, the magnitude and direction of these associations may vary according to the muscle group, assessment method, sport, and population examined [17,18,19]. Although a 10% asymmetry threshold has frequently been used as a practical reference, fixed asymmetry thresholds are context-dependent and should not be interpreted as universally established clinical, injury-risk, or performance cut-offs, as their relevance may vary according to the population, muscle group, and assessment method [19].

Despite the growing body of literature investigating complex training, previous studies have primarily focused on its effects on athletic performance [16,20,21] or injury-related inter-limb asymmetries [22,23]. However, the existing evidence remains inconsistent, partly because of substantial methodological heterogeneity across studies. While several investigations have reported significant improvements in sprint and jump performance following complex training [9,24,25], others have found no meaningful changes in vertical jump performance [26] or repeated sprint ability [27]. Similarly, although some studies have suggested that unilateral complex training is superior to bilateral training for improving sprint and jump performance [25,28], others have reported no significant differences between the two training approaches [15,29].

Moreover, although the effects of complex training on athletic performance have been extensively investigated, relatively few studies have directly examined its influence on lower-limb isokinetic strength, muscle balance, and inter-limb strength asymmetry [23]. Consequently, there remains limited evidence regarding the simultaneous effects of unilateral complex training on isokinetic strength, hamstring-to-quadriceps (H/Q) ratio, inter-limb strength asymmetry, and sport-specific performance in competitive basketball players.

Importantly, the existing knowledge gap extends beyond the limited number of studies investigating unilateral complex training. Most previous research has examined either performance outcomes (e.g., sprint and jump performance) or asymmetry measures in isolation, whereas comprehensive evaluations integrating isokinetic strength, H/Q ratio, bilateral strength asymmetry, and functional performance within the same randomized controlled intervention are scarce [23]. To the best of our knowledge, no previous randomized controlled trial has simultaneously examined the effects of unilateral complex training on lower-limb isokinetic strength, H/Q ratio, bilateral strength asymmetry, and basketball-specific physical performance in competitive young male basketball players. Such a multidimensional approach may provide a more comprehensive understanding of changes in lower-limb strength, inter-limb strength asymmetry, and athletic performance following unilateral complex training. Therefore, the present study was designed to address this important gap by providing an integrated evaluation of these outcomes within a single intervention framework.

Based on these considerations, the primary aim of this randomized controlled trial was to investigate the effects of an eight-week unilateral complex training program on lower-limb isokinetic strength, inter-limb strength asymmetry, hamstring-to-quadriceps (H/Q) ratio, and selected athletic performance outcomes in competitive young male basketball players. We hypothesized that, compared with regular basketball training alone, the addition of an eight-week unilateral complex training program would be associated with greater improvements in lower-limb isokinetic strength, reductions in inter-limb strength asymmetry, improvements in the H/Q ratio, and enhanced sprint and jump performance. This hypothesis was based on evidence suggesting that repeated unilateral loading may promote limb-specific neuromuscular adaptations and contribute to improvements in inter-limb strength balance [30,31]. Furthermore, the repeated combination of resistance and plyometric exercises may promote chronic neuromuscular adaptations, including improvements in force production and stretch-shortening cycle function [32,33]. These chronic adaptations should be distinguished from the acute PAPE response that may occur within individual complex training sessions. Accordingly, the chronic adaptations observed following the eight-week intervention are likely multifactorial and should not be attributed solely to PAPE, but may reflect the cumulative effects of repeated resistance and plyometric loading and associated neuromuscular adaptations.

## 2. Materials and Methods

### 2.1. Study Design

**Figure 1:**
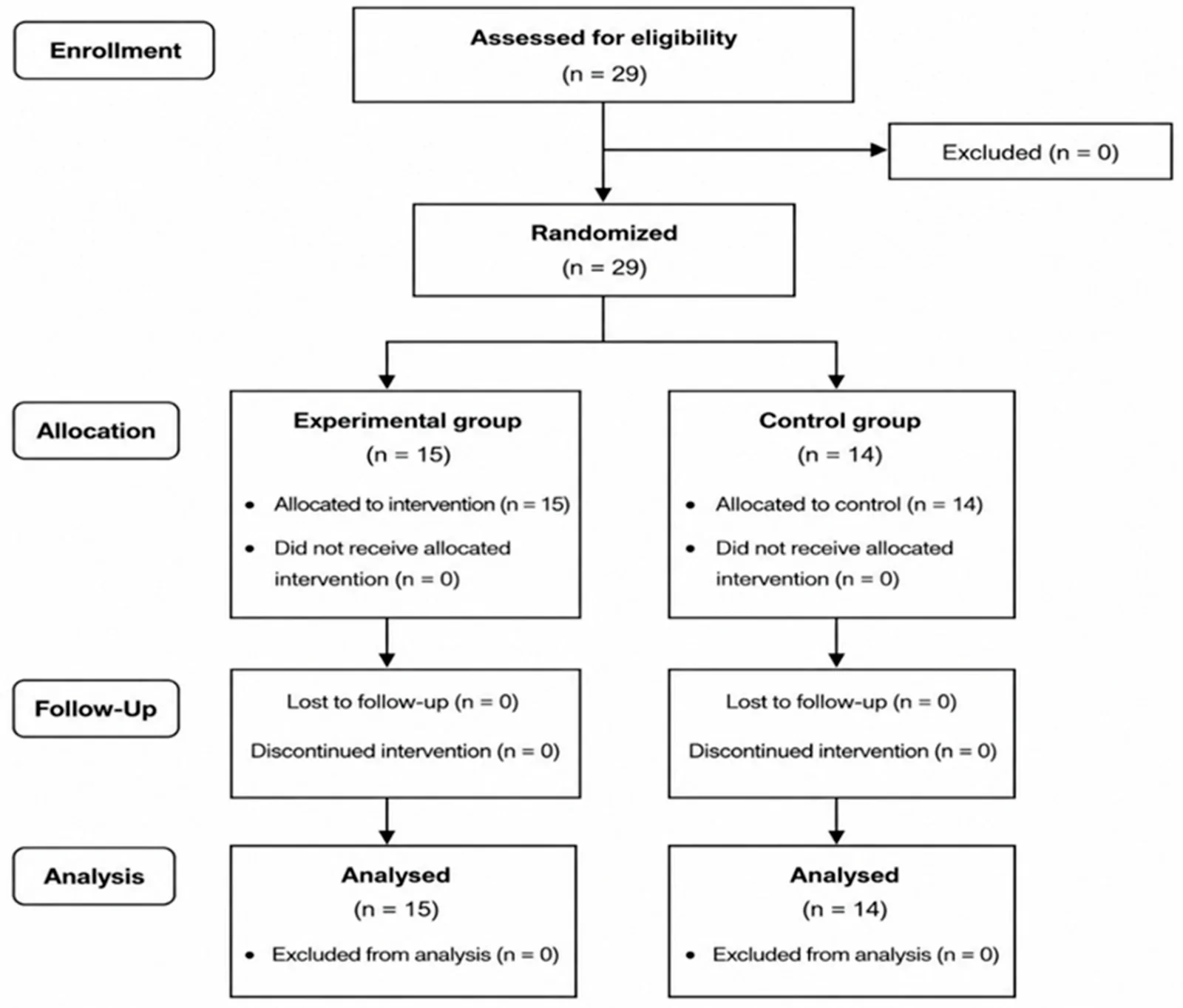
CONSORT Flow Diagram.

This study was designed as a parallel-group, randomized controlled trial with a pretest–posttest design to investigate the effects of an eight-week unilateral complex training program on lower-limb isokinetic strength, inter-limb strength asymmetry, hamstring-to-quadriceps (H/Q) ratio, and athletic performance in competitive young male basketball players. Participants were randomly assigned to either an experimental group or a control group and completed identical assessment protocols before and after the intervention. The random allocation sequence was generated using a computer-based randomization procedure (Randomizer.org) by an independent researcher who was not involved in participant assessment or delivery of the intervention. The same independent researcher implemented the allocation and assigned participants to the experimental or control group in a 1:1 ratio. Allocation concealment was maintained by restricting access to the randomization sequence to the independent researcher; investigators involved in participant enrollment and assessment had no access to the allocation sequence before group assignment. Outcome assessors responsible for the pre- and post-intervention measurements were blinded to group allocation throughout the study. The participant flow through the study is presented in Figure 1. The trial was registered at ClinicalTrials.gov (Identifier: NCT07766096). The primary outcome measures were lower-limb isokinetic strength, inter-limb strength asymmetry, and the H/Q ratio, whereas secondary outcome measures included countermovement jump (CMJ), Abalakov jump, 20-m sprint performance, and force-platform-derived jump variables. The study was conducted and reported in accordance with the Consolidated Standards of Reporting Trials (CONSORT) Statement for randomized controlled trials. The study protocol was reviewed and approved by the Mersin University Sports Sciences Ethics Committee (Approval No. 2026/037; approval date: 4 May 2026) before participant recruitment. All procedures were conducted in accordance with the ethical principles of the Declaration of Helsinki. Prior to participation, all athletes received detailed verbal and written information regarding the study procedures, potential risks, and expected benefits, and provided written informed consent before enrollment.

### 2.2. Participants

Twenty-nine competitive male basketball players (age: 19.14 ± 0.99 years; height: 189.62 ± 7.32 cm; body mass: 79.80 ± 7.27 kg; BMI: 22.15 ± 1.73 kg·m^−2^) from the same basketball club voluntarily participated in this study. The participants competed in two competition categories within the same club, namely the Turkish Basketball Youth League (BGL) and the U18 League, and were considered to have a comparable competitive and training level. Participants from both competition categories were combined into a single participant pool before randomization, and allocation was performed without stratification by competition category. According to the participant classification framework proposed by McKay et al. [34], all athletes were classified as Tier 3 (highly trained/national-level athletes), as they competed in organized national youth competitions and participated in structured basketball training throughout the competitive season. During the study period, all participants completed four basketball-specific training sessions per week and competed in one official league match each weekend. Training sessions followed a standardized team-based program consisting of technical skill development (passing, shooting, dribbling, and lay-up drills), tactical training (offensive and defensive systems), and basketball-specific conditioning, including sprinting, jumping, and change-of-direction exercises [34]. The required sample size was estimated a priori using G*Power (v3.1.9.7, Heinrich Heine University, Düsseldorf, Germany).

The effect-size estimate was derived from a previous intervention study by Gonzalo-Skok et al. [35], which reported a large effect for between-limb imbalance. Based on this evidence, an effect size of Cohen’s f = 0.58 was used for the a priori power analysis. The analysis was specified for a repeated-measures ANOVA (within–between interaction), with α = 0.05, statistical power = 0.80, two groups, two measurements, a correlation of 0.50 among repeated measures, and a nonsphericity correction of 1. The analysis indicated a minimum total sample size of 10 participants. Therefore, the final sample of 29 participants (experimental group: *n* = 15; control group: *n* = 14) exceeded the minimum required sample size. Therefore, the final sample of 29 participants (experimental group: *n* = 15; control group: *n* = 14) was considered sufficient to detect moderate-to-large intervention effects. Participants were randomly allocated to either the experimental or control group using a computer-generated randomization procedure (Randomizer.org). Eligibility criteria included: (a) voluntary participation; (b) a minimum of five years of competitive basketball experience; (c) regular basketball training at least four times per week during the previous two years; and (d) the absence of acute or chronic musculoskeletal injury or medical conditions that could affect basketball performance or participation, as confirmed using the Physical Activity Readiness Questionnaire (PAR-Q); and (e) no participation in a structured unilateral resistance or complex training program during the previous three months. Eligibility was verified through participant self-report and confirmation by each team’s coaching staff. No formal injury surveillance system was implemented during the intervention; however, no participant reported any injury or prolonged absence from training throughout the study period. To minimize potential confounding factors, all participants resided in the same athlete dormitory and followed standardized nutrition programs prepared by a registered sports nutritionist. Meals were identical for all athletes throughout the intervention. Participants were instructed to maintain their habitual lifestyle, refrain from using ergogenic aids, stimulants, or alcohol, and avoid resistance exercise or vigorous physical activity for at least 48 h before each testing session.

### 2.3. Experimental Procedures

**Figure 2:**
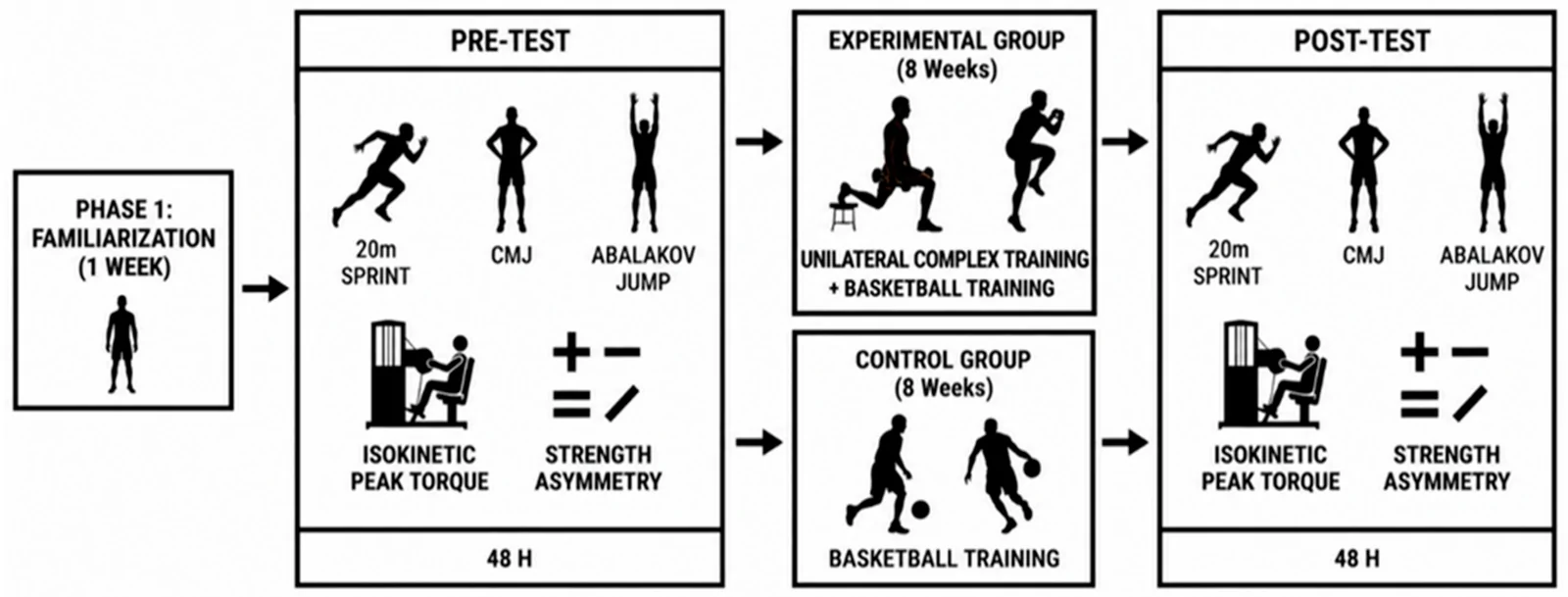
Experimental Design.

The study was conducted in three consecutive phases: (1) baseline assessments (pre-test), (2) an eight-week training intervention, and (3) post-intervention assessments (post-test) (Figure 2). Before baseline testing, all participants completed two familiarization sessions to minimize learning effects and ensure consistent execution of all testing procedures. Each session began with a standardized 15-min dynamic warm-up, consisting of 5 min of light jogging followed by dynamic mobility and activation exercises, including high knees, butt kicks, walking lunges, lateral lunges, leg swings, arm circles, and body-weight squats.

During the first familiarization session, participants received standardized instructions regarding all testing procedures and performed practice trials of the countermovement jump (CMJ), Abalakov jump, 20-m sprint, and isokinetic strength assessments until they demonstrated correct technique and consistent performance. This familiarization procedure was implemented to improve measurement reliability and minimize potential learning effects during baseline testing. The second familiarization session was performed to individualize the resistance loads used during the unilateral complex training program. One-repetition maximum (1RM) for the Bulgarian Split Squat (BSS) was determined according to the National Strength and Conditioning Association (NSCA) Guidelines [36]. Following a standardized warm-up, participants completed progressive loading sets with 1–2 repetitions per attempt and 3–5 min of passive recovery between attempts until the maximum load that could be lifted with proper technique was identified. During testing, the rear foot was positioned on an elevated support while the front leg generated the movement through unilateral force production, with approximately 90° of knee flexion considered the standardized movement criterion [25,37].

Because of the greater balance and coordination demands of the Single-Leg Reverse Lunge and Unilateral Deadlift, direct 1RM testing was not performed for these exercises. Instead, training intensity was prescribed using the 10-point Rating of Perceived Exertion (RPE) scale, with exercise loads adjusted to achieve the target RPE values recommended by Helms et al. [38]. To minimize residual fatigue, all performance assessments were separated by at least 48 h. Baseline testing was performed over three testing sessions. During the first session, body composition was assessed using bioelectrical impedance analysis (BIA), followed by the 20-m sprint test using an electronic timing system. Forty-eight hours later, vertical jump performance was evaluated using bilateral countermovement jump (CMJ) and Abalakov jump tests performed on a Hawkin Dynamics (Hawkin Dynamics, Westbrook, ME, USA). force platform. Following another 48-h recovery period, lower-limb isokinetic strength and strength asymmetry were assessed using a Humac Norm (Cybex II Norm, CSMi, Stoughton, MA, USA) isokinetic dynamometer. After completion of the baseline assessments, participants were randomly allocated to either the experimental group (*n* = 15) or the control group (*n* = 14). The experimental group completed the eight-week unilateral complex training program in addition to their regular basketball training, whereas the control group continued their routine basketball training without any additional intervention. Following the intervention, all outcome measures were repeated using identical testing procedures, equipment, investigators, and environmental conditions. To minimize potential circadian variation, all assessments were performed in the laboratory between 13:00 and 16:00 h. Each participant was assessed at the same time of day during the pre- and post-intervention testing sessions. Throughout the eight-week intervention, participants in both groups continued their habitual basketball schedule, consisting of four team-training sessions and one competitive match per week. However, habitual basketball training load was not quantitatively monitored using internal or external load measures (e.g., session-RPE, heart rate, or GPS-derived metrics). The overall experimental design is illustrated in Figure 2.

### 2.4. Training Intervention

#### 2.4.1. Eight-Week Unilateral Complex Training Program

The unilateral complex training program was developed based on the complex training model proposed by Carter and Greenwood [39], which involves the sequential pairing of a high-intensity unilateral resistance exercise with a biomechanically similar unilateral plyometric exercise. Exercise selection and session organization were adapted from previous unilateral complex training studies [15,25,39,40] and modified to meet the physiological and biomechanical demands of competitive basketball. The intervention was performed twice weekly (Mondays and Thursdays) for eight consecutive weeks, with a minimum recovery period of 72 h between sessions [25]. Each training session lasted approximately 45–55 min. During the intervention period, participants in the control group continued their regular basketball training without performing any additional resistance or plyometric training. Each training session consisted of three unilateral complex pairs, with each pair comprising a unilateral resistance exercise immediately followed by a biomechanically corresponding unilateral plyometric exercise [39]. The resistance exercises included the Bulgarian Split Squat, Single-Leg Reverse Lunge, and Unilateral Deadlift, whereas the corresponding plyometric exercises consisted of the Rear-Foot Elevated Split Jump, Single-Leg Vertical Jump, and Single-Arm Alternate-Leg Bound, respectively. Following completion of each resistance exercise, participants rested passively for 2 min before performing the paired plyometric exercise. A 3-min passive recovery period was provided between successive complex pairs. These recovery intervals were selected according to previous recommendations for optimizing the post-activation performance enhancement (PAPE) response during complex training [39]. These recovery intervals were adapted from general complex training recommendations and were not specifically validated for unilateral exercise configurations. To ensure balanced lower-limb development, the program incorporated not only quadriceps-dominant exercises but also unilateral hip- and hamstring-dominant movements targeting the posterior kinetic chain, which plays a fundamental role in sprinting, jumping, deceleration, and change-of-direction performance in basketball. Consequently, the program was specifically designed to improve both knee extensor and knee flexor strength, optimize the hamstring-to-quadriceps (H/Q) ratio, and reduce inter-limb strength asymmetry while simultaneously enhancing basketball-specific athletic performance. All training sessions were supervised by certified strength and conditioning specialists, who monitored exercise technique, training intensity, adherence to the prescribed protocol, and participant safety throughout the intervention. Prior to the intervention, limb dominance was determined individually using two self-reported functional questions. Participants were asked (1) which leg they preferentially used when shooting a basketball and (2) which leg they used most frequently during sport-related activities. The two questions were used to cross-confirm functional limb preference, and concordant responses were considered to indicate the participant’s dominant limb. The contralateral limb was classified as the non-dominant limb. Individual responses were recorded before the intervention and subsequently used to prescribe limb-specific training volume. Accordingly, the dominant limb performed three sets, whereas the non-dominant limb performed five sets of the prescribed unilateral exercises throughout the intervention. A detailed overview of the exercise selection, training loads, progression, and weekly program structure is presented in Table 1. The unequal 3:5 set prescription was intentionally adopted to provide a greater training stimulus to the functionally defined non-dominant limb while maintaining training exposure for both limbs. The 3:5 set prescription was applied to each exercise. Resistance exercises were performed for 5 repetitions per set and plyometric exercises for 10 repetitions per set. Across the two weekly sessions, this corresponded to 24 sets and 180 repetitions for the dominant limb and 40 sets and 300 repetitions for the non-dominant limb per week.

#### 2.4.2. Training Load Progression

Training loads were progressively increased throughout the intervention to ensure progressive overload while maintaining proper exercise technique. For the Bulgarian Split Squat, external loads were prescribed according to each participant’s 1RM and adjusted according to the progression presented in Table 1. Because direct 1RM testing was not appropriate for the Single-Leg Reverse Lunge and Unilateral Deadlift due to their greater balance and coordination demands, training intensity for these exercises was regulated using the 10-point Rating of Perceived Exertion (RPE) scale [36]. Exercise loads were adjusted whenever participants consistently reported RPE values below the prescribed target range. This approach ensured individualized progression while maintaining the intended training stimulus throughout the eight-week intervention.

### 2.5. Outcome Measures

All outcome assessments were conducted in the Physical Profile, Performance and Biomechanics Laboratory, Faculty of Sports Sciences, Mersin University, by the same investigators using standardized testing procedures. These investigators remained blinded to participants’ group allocation during both pre- and post-intervention assessments. Baseline and post-intervention assessments were performed under identical environmental conditions and according to the same measurement protocols.

Body Composition: Body composition was assessed using a multi-frequency bioelectrical impedance analyzer (Tanita BC-418, Tanita Corporation, Tokyo, Japan). Participants were instructed to refrain from food and caffeine intake for at least 6 h before testing, avoid strenuous exercise during the preceding 24 h, and maintain their normal hydration status to minimize measurement error. Measurements were performed with participants barefoot and wearing light athletic clothing. Standing height was measured to the nearest 0.1 cm using a wall-mounted stadiometer (Seca GmbH, Hamburg, Germany). Body mass, body mass index (BMI), and body composition variables were obtained according to the manufacturer’s guidelines and standardized anthropometric assessment procedures [41].

20-m Sprint Test: Twenty-meter sprint performance was assessed using a dual-beam electronic timing system (Fusion Sport SmartSpeed, Fusion Sport, Brisbane, Australia). Participants started from a standing position 1 m behind the first timing gate to eliminate premature triggering of the photocells. On the tester’s command, participants sprinted maximally through the finish line without deceleration before passing the second timing gate. Each participant performed three maximal trials, separated by 3 min of passive recovery to minimize fatigue. The fastest sprint time was retained for subsequent statistical analyses. Electronic photocell timing systems have been shown to provide valid and reliable measurements of sprint performance in athletic populations [42,43].

Countermovement Jump and Abalakov Jump Test: Vertical jump performance was assessed using a dual force platform (Hawkin Dynamics Inc., Westbrook, ME, USA) through the Countermovement Jump (CMJ) and Abalakov Jump tests. Before testing, participants received standardized instructions and performed familiarization trials. For both jump tests, participants were instructed to perform a rapid countermovement followed by a maximal vertical jump. During the CMJ, participants kept their hands on their hips throughout the movement to eliminate upper-body contribution, whereas in the Abalakov Jump, unrestricted arm swing was permitted to allow the contribution of upper-limb momentum to jump performance [44]. All jumps were performed bilaterally on the force platform using a self-selected countermovement depth. Each participant completed three maximal trials for each jump test, separated by 60 s of passive recovery, and the highest jump height (cm) was retained for statistical analysis. In addition to jump height, flight time (ms), time to takeoff (ms), and peak force (N) were automatically calculated using the Hawkin Dynamics force platform system (version 10.7.0). These force–time variables provide additional information regarding lower-limb neuromuscular function beyond jump height alone [45]. The Hawkin Dynamics force platform has previously demonstrated excellent validity and reliability for assessing vertical jump performance in athletic populations [44].

Isokinetic Strength Assessment: Participants: Knee flexor and extensor strength was assessed at pre-test and post-test using a Cybex II Norm isokinetic dynamometer (CSMi, Stoughton, MA, USA). Before testing, a standardized warm-up protocol of approximately 15 min was administered to prepare the athletes for the measurement. Participants then completed a familiarization set of 10 repetitions at an angular velocity of 180°·s^−1^ to become accustomed to the device. Isokinetic strength assessment was performed at an angular velocity of 60°·s^−1^, consisting of 3 sets × 5 maximal concentric repetitions. The angular velocity of 60°·s^−1^ was selected because it allows evaluation of maximal isokinetic peak torque and is widely used in the sports science literature for determining inter-limb strength asymmetry, owing to its high measurement reliability. Furthermore, given the greater force output typically observed at lower contraction velocities, this speed enables assessment of maximal strength capacity and improves comparability with previous studies examining strength asymmetry in team-sport athletes [46]. To minimize the risk of hamstring injury during testing, all measurements were conducted exclusively in the concentric/concentric (Con/Con) contraction mode [47]. Participants were seated in the dynamometer chair according to the manufacturer’s standard protocol at a hip flexion angle of 85°, with the trunk and thigh secured using stabilization straps, and the ankle attached to the dynamometer’s lever arm with a padded cuff. Knee joint range of motion was standardized to 90° for all participants [48]. Measurements were conducted in a laboratory environment with stable temperature and humidity conditions, between 13:00 and 16:00. A 60-s passive rest interval was provided between sets to ensure adequate recovery. Participants were informed of the testing procedure beforehand and were encouraged with standardized verbal motivation throughout testing. Peak torque (PT) values obtained from all assessments were recorded in Newton-meters (Nm). The dominant limb was tested first in all participants, followed by the non-dominant limb; therefore, limb-testing order was standardized rather than randomized. For each assessment, the anatomical axis of the knee joint was aligned with the dynamometer axis using the lateral femoral epicondyle as the anatomical reference. Gravity correction was performed according to the manufacturer’s procedures before testing, and the dynamometer was calibrated before each testing session in accordance with the manufacturer’s recommendations. Participants completed three sets of five maximal repetitions at 60°·s^−1^, and the highest peak torque value obtained across the three sets was retained for each limb and movement for statistical analysis.

Calculation of Bilateral/Ipsilateral Strength Asymmetry: To determine lower-extremity strength balance, bilateral and ipsilateral strength asymmetries were calculated. Bilateral strength asymmetry was calculated separately for the quadriceps and hamstring muscle groups using the peak torque values obtained at an angular velocity of 60°·s^−1^. The bilateral asymmetry percentage for the quadriceps (Q/Q) and hamstring (H/H) muscle groups was determined using the following formula:

Bilateral Asymmetry (%) = [(Higher peak torque − Lower peak torque)/Higher peak torque] × 100

This formula was selected because it is one of the calculation approaches widely used in the strength asymmetry literature and allows for a reliable determination of bilateral performance differences obtained from isokinetic assessments. In addition, this approach does not rely on the dominant limb or a predetermined fixed reference limb; instead, it is based on the absolute performance difference between the two limbs, allowing for a more objective assessment of true inter-limb asymmetry [49]. Ipsilateral strength balance was calculated using the peak torque values of the hamstring and quadriceps muscles for both limbs. The hamstring/quadriceps (H/Q) strength ratio for the right and left legs was expressed as a percentage (%) using the following formula [50]:

H/Q (%) = (Hamstring peak torque/Quadriceps peak torque) × 100

### 2.6. Statistical Analysis

All statistical analyses were performed using IBM SPSS Statistics (Version 27.0; IBM Corp., Armonk, NY, USA) and GraphPad Prism (Version 10.6.1; GraphPad Software, San Diego, CA, USA). Data are presented as mean ± standard deviation (SD). The normality of data distribution was assessed using the Shapiro–Wilk test, while the homogeneity of variances was examined using Levene’s test. Since the within-subject factor (time) had only two levels (pre-test and post-test), the assumption of sphericity was automatically satisfied. Therefore, Mauchly’s test of sphericity and Greenhouse–Geisser corrections were not applied. Baseline differences between the experimental and control groups were assessed using an independent samples *t*-test. To determine the effects of the intervention, a two-way mixed-design ANOVA was applied to all dependent variables, with group (experimental group and control group) treated as the between-subjects factor and time (pre-test and post-test) as the within-subjects factor. When significant interaction effects were observed, pairwise comparisons were conducted using Bonferroni-corrected post hoc analyses. When significant interaction effects were observed, pairwise comparisons were conducted using Bonferroni-corrected post hoc analyses. Given the number of outcome variables examined, lower-limb isokinetic strength and inter-limb strength asymmetry were considered the principal outcomes of the study, whereas H/Q ratios and physical performance variables were treated as secondary/exploratory outcomes. The Bonferroni adjustment was applied to simple-effects/pairwise comparisons following significant Group × Time interactions but was not applied across the separate outcome-specific ANOVAs. Accordingly, findings for the secondary/exploratory outcomes were interpreted cautiously, with emphasis on effect sizes and 95% confidence intervals alongside *p*-values. It should be noted that a statistically significant Group × Time interaction indicates that the trend of change over time differs between groups, but does not by itself specify the direction of this difference (e.g., greater improvement in the experimental group, a decline in the control group, or both). To clarify the direction and magnitude of change within each group, within-group pre-to-post effect sizes were calculated as Cohen’s dz for paired measurements, defined as the mean of the individual pre-to-post difference scores divided by the standard deviation of the corresponding difference scores (dz = Mean Difference/SD difference). Effect sizes were calculated using partial eta squared (ηp^2^) and interpreted according to the following threshold values: small (ηp^2^ ≥ 0.01), medium (ηp^2^ ≥ 0.06), and large (ηp^2^ ≥ 0.14). Statistical significance for all analyses was set at *p* < 0.05.

## 3. Results

The baseline demographic, anthropometric, and outcome characteristics of the participants are presented in Table 2. No statistically significant between-group differences were observed at baseline for demographic, anthropometric, strength, asymmetry, or physical performance variables (all *p* > 0.05). Notably, the baseline difference in Flight Time between the experimental and control groups was not statistically significant (*p* = 0.137). Baseline comparisons are provided to characterize the randomized groups and were not interpreted as evidence of statistical equivalence.

**Figure 3:**
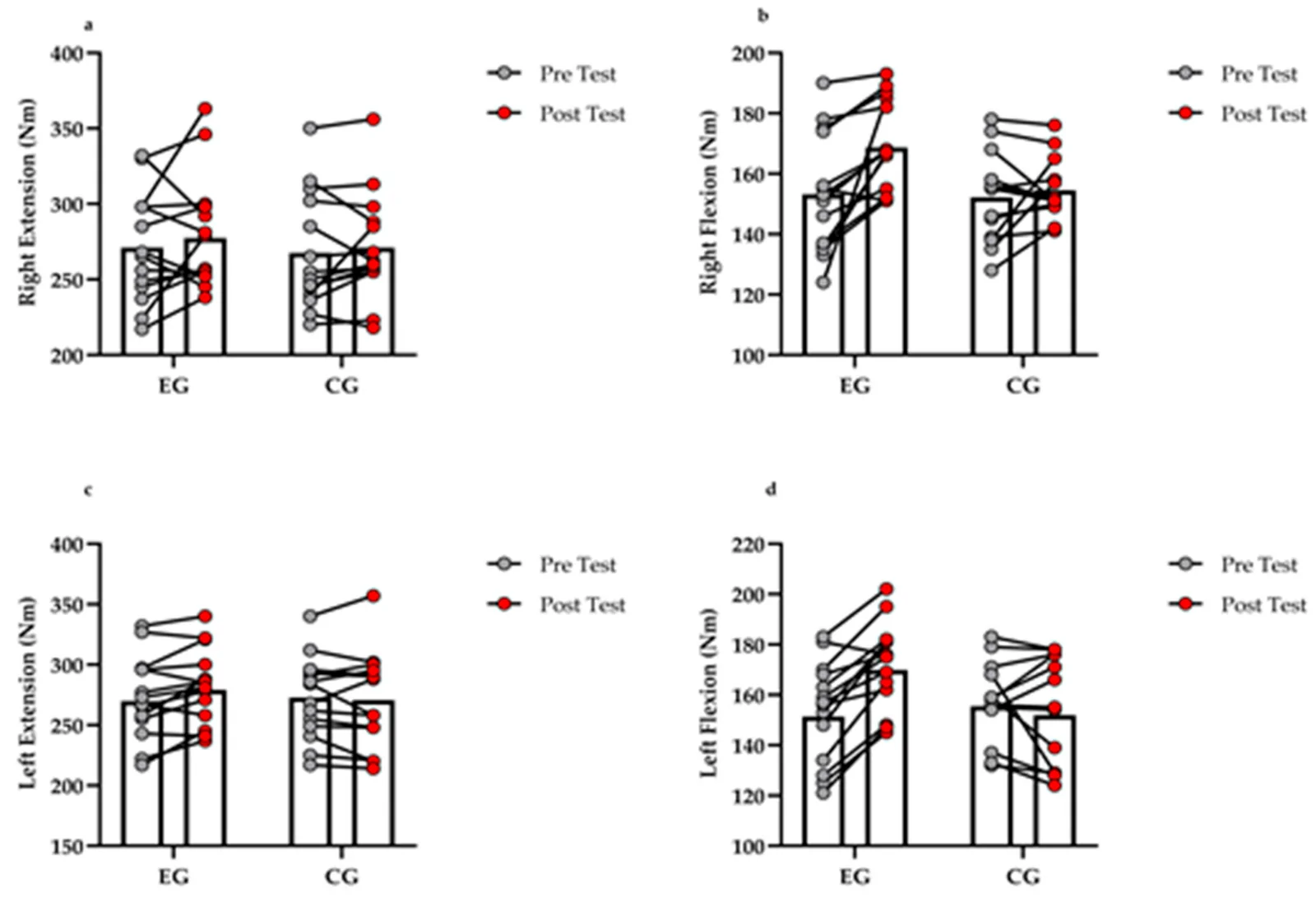
Pre- and Post-Intervention Isokinetic Strength Variables in the Experimental and Control Groups: (**a**) right knee extension; (**b**) right knee flexion; (**c**) left knee extension; (**d**) left knee flexion.

**Figure 4:**
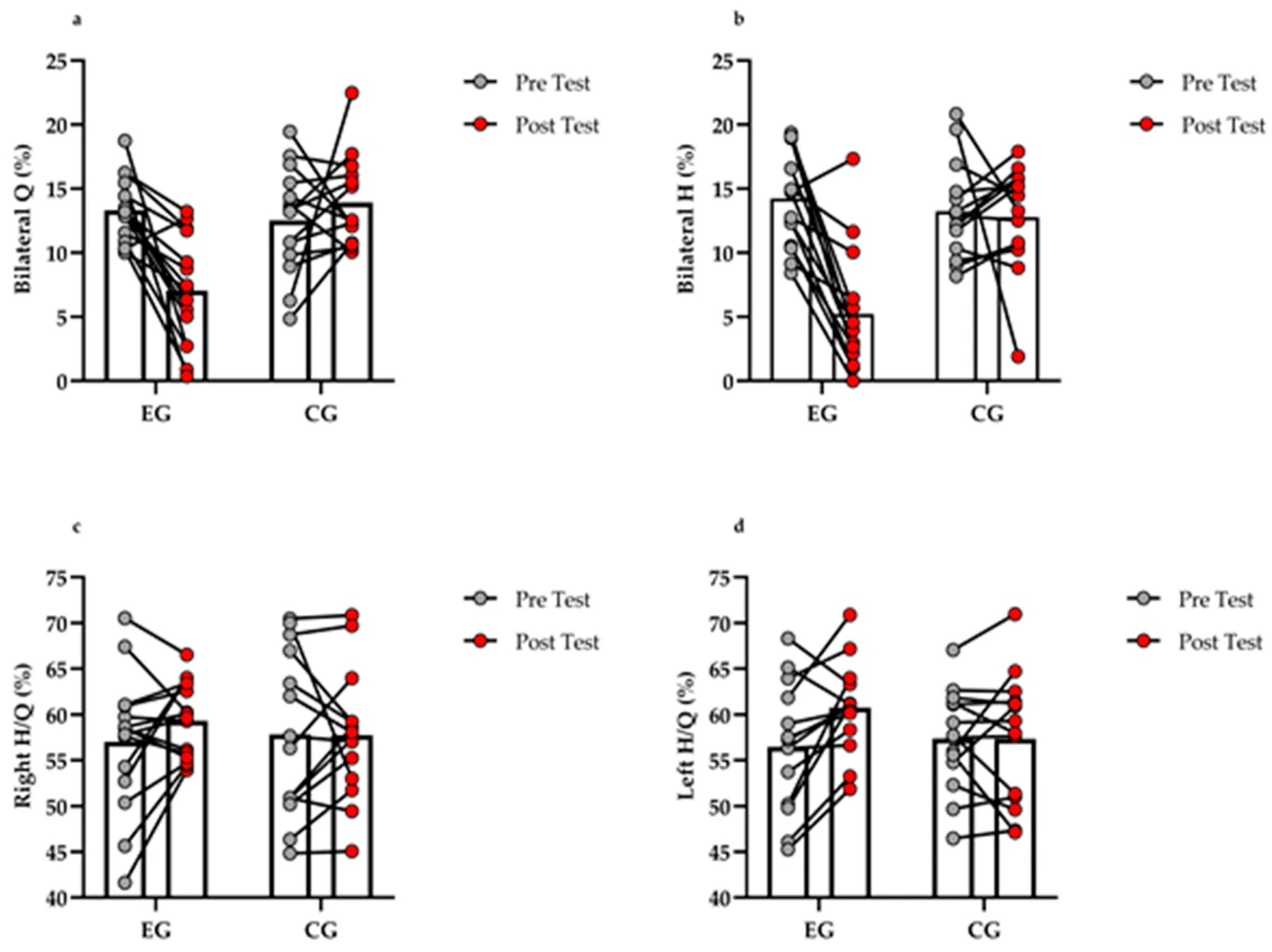
Pre- and Post-Intervention Lower Extremity Asymmetry Variables in the Experimental and Control Groups: (**a**) bilateral quadriceps strength asymmetry (Bilateral Q); (**b**) bilateral hamstring strength asymmetry (Bilateral H); (**c**) right hamstring-to-quadriceps ratio (Right H/Q); (**d**) left hamstring-to-quadriceps ratio (Left H/Q).

Table 3 presents the two-way mixed-design ANOVA results for isokinetic strength variables (Figure 3). Significant main effects of time were observed for right knee extension, right knee flexion, and left knee flexion, with large effect sizes, whereas the time effect for left knee extension was not significant. No significant main effects of group were observed. Significant Group × Time interactions, all with large effect sizes, were found for right knee flexion, left knee extension, and left knee flexion, whereas the interaction for right knee extension was not significant. Detailed statistics, including *p* values, ηp^2^, and 95% confidence intervals, are presented in Table 3. Bonferroni-adjusted simple-effects analyses showed significant pre-to-post improvements in the experimental group for right knee flexion (*p* = 0.001), left knee extension (*p* = 0.007), and left knee flexion (*p* = 0.001), whereas no significant changes were observed in the control group (all *p* > 0.05). Post-intervention between-group differences were significant for right knee flexion (*p* = 0.006) and left knee flexion (*p* = 0.036), but not for left knee extension (*p* = 0.526) (Table S1). Table 4 presents the two-way mixed-design ANOVA results for the lower-extremity asymmetry variables (Figure 4). A significant main effect of time was found for bilateral quadriceps asymmetry (*p* = 0.018, ηp^2^ = 0.19, large effect, 95% CI = 0.00–0.42), bilateral hamstring asymmetry (*p* = 0.001, ηp^2^ = 0.42, large effect, 95% CI = 0.14–0.61), and left H/Q ratio (*p* = 0.024, ηp^2^ = 0.18, large effect, 95% CI = 0.00–0.40), whereas no significant time effect was observed for right H/Q ratio (*p* = 0.352, ηp^2^ = 0.03, small effect, 95% CI = 0.00–0.23). Regarding the main effect of group, significant effects were found for bilateral quadriceps asymmetry (*p* = 0.004, ηp^2^ = 0.29, large effect, 95% CI = 0.03–0.49) and bilateral hamstring asymmetry (*p* = 0.006, ηp^2^ = 0.25, large effect, 95% CI = 0.02–0.47), whereas no significant group effect was found for right H/Q ratio (*p* = 0.869, ηp^2^ = 0.01, small effect, 95% CI = 0.00–0.10) or left H/Q ratio (*p* = 0.553, ηp^2^ = 0.01, small effect, 95% CI = 0.00–0.18). The Group × Time interaction was significant for bilateral quadriceps asymmetry, bilateral hamstring asymmetry, and left H/Q ratio (*p* = 0.001, ηp^2^ = 0.37, large effect, 95% CI = 0.09–0.57; *p* = 0.001, ηp^2^ = 0.38, large effect, 95% CI = 0.10–0.57; and *p* = 0.023, ηp^2^ = 0.18, large effect, 95% CI = 0.00–0.41, respectively). In contrast, no significant interaction effect was observed for right H/Q ratio (*p* = 0.318, ηp^2^ = 0.04, small effect, 95% CI = 0.00–0.24) (*p* > 0.05). Bonferroni-adjusted simple-effects analyses showed significant pre-to-post changes in the experimental group for bilateral quadriceps asymmetry (*p* = 0.001), bilateral hamstring asymmetry (*p* = 0.001), and left H/Q ratio (*p* = 0.002), whereas no significant changes were observed in the control group (all *p* > 0.05). Post-intervention between-group differences were significant for bilateral quadriceps and hamstring asymmetry (both *p* = 0.001), but not for the left H/Q ratio (*p* = 0.140) (Table S1). Because the magnitude-based asymmetry index does not retain the direction of asymmetry, an additional individual-level analysis was performed to examine the identity and consistency of the stronger and weaker limbs. At baseline, the non-dominant limb showed lower quadriceps peak torque in all participants in the experimental group (15/15) and control group (14/14), and lower hamstring peak torque in 12/15 and 12/14 participants, respectively. From pre- to post-intervention, the identity of the stronger/weaker limb remained unchanged for quadriceps peak torque in 12/15 participants in the experimental group and 14/14 participants in the control group, and for hamstring peak torque in 11/15 and 12/14 participants, respectively. Thus, the weaker-limb designation was largely, but not universally, consistent across testing occasions.

**Figure 5:**
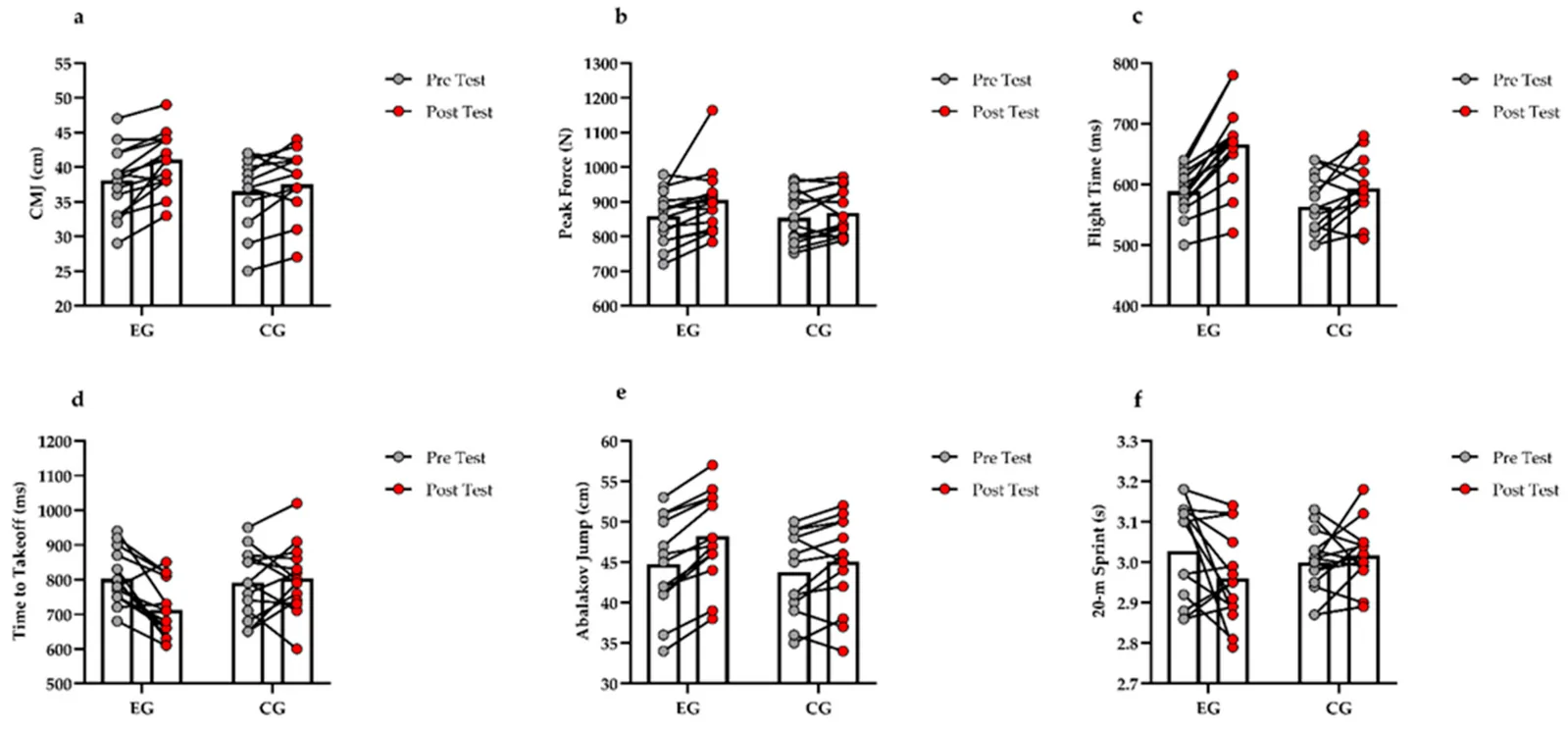
Pre- and Post-Intervention Physical Performance Variables in the Experimental and Control Groups: (**a**) countermovement jump (CMJ); (**b**) peak force; (**c**) flight time; (**d**) time to takeoff; (**e**) Abalakov jump; (**f**) 20-m sprint.

The two-way mixed-design ANOVA results for participants’ physical performance variables are presented in Table 5 and Figure 5. A significant main effect of time was found for CMJ (*p* = 0.001, ηp^2^ = 0.40, large effect, 95% CI = 0.12–0.59), Peak Force (*p* = 0.003, ηp^2^ = 0.28, large effect, 95% CI = 0.04–0.50), Flight Time (*p* = 0.001, ηp^2^ = 0.65, large effect, 95% CI = 0.39–0.76), Time to Takeoff (*p* = 0.013, ηp^2^ = 0.21, large effect, 95% CI = 0.01–0.44), and Abalakov Jump (*p* = 0.001, ηp^2^ = 0.64, large effect, 95% CI = 0.38–0.76), whereas no significant effect was observed for 20-m Sprint (*p* = 0.202, ηp^2^ = 0.06, medium effect, 95% CI = 0.00–0.27). A significant main effect of group was found for Flight Time (*p* = 0.010, ηp^2^ = 0.22, large effect, 95% CI = 0.01–0.44) (*p* < 0.05), while no significant group effect was observed for the other variables (*p* > 0.05). The Group × Time interaction was significant for CMJ (*p* = 0.045, ηp^2^ = 0.14, large effect, 95% CI = 0.00–0.37), Flight Time (*p* = 0.005, ηp^2^ = 0.26, large effect, 95% CI = 0.03–0.48), Time to Takeoff (*p* = 0.002, ηp^2^ = 0.31, large effect, 95% CI = 0.06–0.52), Abalakov Jump (*p* = 0.003, ηp^2^ = 0.28, large effect, 95% CI = 0.04–0.50), and 20-m Sprint (*p* = 0.026, ηp^2^ = 0.17, large effect, 95% CI = 0.00–0.40). No significant interaction effect was found for Peak Force (*p* = 0.099, ηp^2^ = 0.10, medium effect, 95% CI = 0.00–0.32). Bonferroni-adjusted simple-effects analyses showed significant pre-to-post changes in the experimental group for CMJ (*p* = 0.001), Flight Time (*p* = 0.001), Time to Takeoff (*p* = 0.001), Abalakov Jump (*p* = 0.001), and 20-m Sprint (*p* = 0.014). In the control group, significant pre-to-post changes were observed for Flight Time (*p* = 0.011) and Abalakov Jump (*p* = 0.014), whereas changes in CMJ, Time to Takeoff, and 20-m Sprint were not significant (all *p* > 0.05). Post-intervention between-group differences were significant for CMJ (*p* = 0.041), Flight Time (*p* = 0.003), and Time to Takeoff (*p* = 0.011), but not for Abalakov Jump (*p* = 0.132) or 20-m Sprint (*p* = 0.109) (Table S1).

## 4. Discussion

The primary finding of the present study was that eight weeks of unilateral complex training significantly improved lower-limb muscle function, reduced inter-limb strength asymmetry, and enhanced athletic performance in competitive young male basketball players. Specifically, greater reductions were observed in bilateral quadriceps (−47.07%) and hamstring (−63.41%) strength asymmetry, accompanied by a significant improvement in the left hamstring-to-quadriceps (H/Q) ratio. Furthermore, significant increases were observed in right knee flexor, left knee flexor, and left knee extensor isokinetic strength compared with the control group. Collectively, these findings suggest that the addition of unilateral complex training to regular basketball practice was associated with improvements in lower-limb neuromuscular function and inter-limb strength balance. The intervention was associated with limb-specific changes in isokinetic strength, with significant Group × Time interactions observed for right knee flexion, left knee extension, and left knee flexion. However, functional limb dominance should not be equated with baseline strength, and the present findings do not establish that the left limb consistently represented either the non-dominant or weaker limb across participants. Therefore, these side-specific findings are interpreted in terms of right- and left-limb responses rather than as preferential adaptations of the weaker or non-dominant limb. The observed improvements in isokinetic strength and reductions in inter-limb strength asymmetry are consistent with previous studies reporting superior neuromuscular adaptations following unilateral resistance and complex training [13,25]. Although most previous investigations have primarily examined sprint and jump performance, relatively few have evaluated changes in isokinetic muscle strength and strength asymmetry simultaneously. Therefore, the present findings extend the existing literature by showing that the addition of unilateral complex training to regular basketball practice was associated with improvements in selected athletic performance outcomes and lower-limb strength balance. Compared with bilateral exercises, unilateral loading may be associated with greater neural drive and motor unit recruitment, which could potentially influence neural constraints related to the bilateral deficit phenomenon [30,31]. Such neural adaptations may partly contribute to changes in lower-limb strength; however, neural activation was not directly assessed in the present study, and these mechanisms should therefore be considered speculative.

Regarding the H/Q ratio, a significant Group × Time interaction was observed only for the left limb, whereas no significant interaction was found for the right limb, indicating a side-specific response. Although the intervention incorporated both quadriceps- and posterior-chain-oriented unilateral exercises, the multi-component nature of the training program does not allow the observed change in the left H/Q ratio to be attributed to any individual exercise or training component. Therefore, the improvement in the left H/Q ratio should be interpreted as an adaptation associated with the overall intervention. Furthermore, because muscle architecture and musculotendinous properties were not directly assessed, the physiological mechanisms underlying this side-specific response remain speculative [51]. The present findings are consistent with the growing body of evidence supporting the effectiveness of unilateral training interventions for improving lower-limb strength and reducing inter-limb asymmetry. Parlak et al. [26] reported that a 12-week resistance and plyometric training program significantly increased isokinetic muscle strength at multiple angular velocities in young male basketball players, supporting the improvements in peak torque observed in the present study. Similarly, Zhang et al. [23] demonstrated that a 10-week targeted unilateral compound training program enhanced maximal lower-limb strength while significantly reducing inter-limb asymmetry in elite collegiate basketball players. These findings are further corroborated by Güven et al. [52], who reported that an unilateral compound training intervention significantly reduced lower-limb strength asymmetry and enhanced athletic performance in female handball players, suggesting that the beneficial effects of unilateral loading on inter-limb symmetry may generalize across different team-sport populations and sexes. Comparable findings were also reported by Duan et al. [28], who observed significant improvements in sprint performance, change-of-direction ability, and inter-limb asymmetry following an eight-week unilateral contrast training program in university basketball players. Collectively, these findings suggest that unilateral loading strategies consistently promote favorable neuromuscular adaptations in basketball athletes. The substantial reductions in inter-limb strength asymmetry observed in the present study may be attributed to the asymmetric loading characteristics of the training program. Because unilateral exercises require each limb to generate force independently, the weaker or non-dominant limb is exposed to a relatively greater training stimulus, thereby facilitating greater neuromuscular adaptations and narrowing strength deficits between limbs [23]. This explanation is supported by the greater improvements observed in the non-dominant limb, particularly for left knee flexor strength. Despite the increasing popularity of unilateral complex training, relatively few studies have simultaneously examined lower-limb isokinetic strength, inter-limb strength asymmetry, and muscle balance within the same intervention. Moreover, previous investigations have predominantly focused on jump-based asymmetry assessments [53,54], whereas fewer studies have employed isokinetic dynamometry to quantify strength asymmetry. Because isokinetic dynamometry is considered one of the most reliable methods for evaluating muscle strength and inter-limb imbalances, its inclusion provides a more comprehensive assessment of neuromuscular function [51].

The present study also demonstrated that eight weeks of unilateral complex training produced significant improvements in several measures of athletic performance. Compared with the control group, participants in the experimental group showed significant increases in countermovement jump (CMJ) height (+13.67%), flight time (+13.13%), and Abalakov jump performance (+7.75%), together with a significant improvement in 20-m sprint performance (−2.31%). In addition, time to takeoff decreased by 11.30%, indicating a shorter overall duration from the initiation of the countermovement to takeoff. In contrast, although peak force increased by 5.33% in the experimental group, the Group × Time interaction did not reach statistical significance. These findings are consistent with previous studies demonstrating that unilateral complex training enhances explosive performance in basketball players and other athletic populations [15,25,28]. The simultaneous improvements in jump and sprint performance may be explained by the combined effects of unilateral resistance and plyometric exercises, which target both maximal force production and rapid force application through movement patterns that closely resemble sport-specific actions. Such adaptations are considered particularly relevant for basketball, where repeated sprinting, jumping, and rapid changes of direction are fundamental performance determinants. Interestingly, changes were observed in temporal characteristics of jump performance, including flight time and time to takeoff, whereas the Group × Time interaction for peak force was not statistically significant. These findings indicate changes in overall jump-performance characteristics; however, because the force–time curve was not segmented into eccentric and concentric phases, the specific phase-related mechanisms underlying these changes cannot be determined from the present data. One plausible explanation is that repeated exposure to unilateral resistance–plyometric exercise pairs enhanced neuromuscular coordination and the utilization of the stretch-shortening cycle (SSC), thereby facilitating faster force transmission during explosive movements. Although rate of force development (RFD) and SSC function were not directly assessed in the present study, these mechanisms have been proposed as potential contributors to the performance improvements observed following complex training interventions [32,33].

The concurrent improvements in sprint and jump performance observed in the present study may be explained, at least in part, by the neuromuscular adaptations associated with post-activation performance enhancement (PAPE). During complex training, a high-intensity unilateral resistance exercise is immediately followed by a biomechanically similar plyometric movement, a sequence that has been shown to acutely enhance motor neuron excitability and myosin regulatory light-chain phosphorylation, thereby facilitating subsequent explosive performance [33,55,56]. Similarly, acute conditioning activities performed immediately before explosive tasks have been shown to elicit measurable post-activation performance enhancement responses in team-sport athletes [57], supporting the plausibility of a PAPE-mediated mechanism underlying the present findings. Repeated exposure to this stimulus over an extended training period may promote chronic neuromuscular adaptations that improve explosive movement efficiency. Recent evidence in basketball players has demonstrated that complex conditioning activities not only acutely enhance sprint and jump performance but also induce transient increases in Achilles tendon stiffness [32]. Although tendon mechanical properties were not directly assessed in the present study, repeated exposure to PAPE-inducing stimuli may have contributed to improvements in musculotendinous stiffness and stretch-shortening cycle (SSC) efficiency. Such adaptations could partly explain the observed increases in flight time, reductions in time to takeoff, and improvements in overall jump performance following the intervention. The present findings are consistent with previous investigations reporting positive effects of complex and unilateral training on explosive performance. Santos and Janeira [9] demonstrated that a 10-week complex training program significantly improved sprint performance as well as CMJ and Abalakov jump performance in young male basketball players. More recently, Zhang and Li [58] reported that integrating unilateral plyometric training with linear sprint training produced greater improvements in explosive power, agility, and repeated-sprint performance than bilateral training. Similarly, Wang et al. [59] observed that an eight-week unilateral flywheel-based complex training program elicited superior improvements in short-distance sprint and change-of-direction performance compared with bilateral training in elite male volleyball players. Collectively, these findings support the effectiveness of unilateral complex training as a strategy for enhancing explosive athletic performance across different team sports.

Further evidence supporting the effectiveness of combined resistance and plyometric training has been reported in basketball and other team sports. Yáñez-García et al. [24] demonstrated that a six-week combined resistance and plyometric training program significantly improved maximal strength, CMJ performance, and both 10- and 20-m sprint performance in elite youth basketball players. Similarly, Deng et al. [25] reported that unilateral complex-contrast training elicited greater improvements in CMJ performance than bilateral training in volleyball players. In contrast, Bandasak and Lawsrirat [29] found that although both unilateral and bilateral complex training significantly enhanced jump height and lower-limb maximal strength in male collegiate basketball players, no significant between-group differences were observed for jump performance. Likewise, Biel et al. [15] reported improvements in unilateral jump performance following eight weeks of complex training, whereas bilateral CMJ and change-of-direction performance remained unchanged. These findings collectively suggest that the magnitude of performance adaptations may depend on factors such as training status, exercise selection, intervention duration, and the specific performance outcomes assessed. Supporting this interpretation, Mikołajec et al. [16] demonstrated that altering the sequence of resistance and plyometric exercises produced comparable improvements in vertical jump and sprint performance, suggesting that the neuromuscular stimulus generated by combined loading may be more influential than the exercise order itself. The physiological basis for these adaptations has been attributed to improvements in musculotendinous stiffness, more efficient utilization of elastic energy during the stretch-shortening cycle (SSC), enhanced motor unit recruitment, and improved intermuscular coordination [60]. An additional finding of the present study was the concurrent reduction in inter-limb strength asymmetry together with improvements in sprint and jump performance. Although causality cannot be established, it is plausible that a more balanced distribution of force between the lower limbs contributed to more efficient force production during explosive movements. Previous research has shown that greater inter-limb asymmetries are associated with impaired athletic performance, whereas lower asymmetry levels may facilitate more effective movement mechanics [61]. Therefore, the reductions in bilateral strength asymmetry observed in the present study may have contributed, at least in part, to the improvements in sprinting and jumping performance. One of the principal strengths of the present study lies in its comprehensive evaluation of unilateral complex training within a randomized controlled design. Unlike most previous investigations, which have primarily examined bilateral complex training or focused on isolated performance outcomes, the present study simultaneously evaluated lower-limb isokinetic strength, bilateral and ipsilateral strength asymmetry, the hamstring-to-quadriceps (H/Q) ratio, and basketball-specific performance measures. This multidimensional approach provides a more comprehensive understanding of the neuromuscular adaptations induced by unilateral complex training. From a practical perspective, the unilateral training model adopted in this study closely reflects the movement demands of basketball, which frequently involves unilateral actions such as lay-ups, single-leg jumps, accelerations, decelerations, and rapid changes of direction. Consequently, pairing high-intensity unilateral resistance exercises with biomechanically matched unilateral plyometric exercises may represent a sport-specific training strategy capable of simultaneously improving lower-limb muscle balance and explosive athletic performance. In the present study, reductions in inter-limb strength asymmetry occurred alongside improvements in selected athletic performance outcomes. However, the association between changes in asymmetry and changes in athletic performance was not directly examined. Therefore, these concurrent adaptations should be interpreted as separate findings, and it cannot be determined whether reductions in inter-limb strength asymmetry contributed to the observed improvements in sprint or jump performance.

Collectively, the present findings indicate improvements in selected lower-limb strength outcomes and reductions in the measured inter-limb strength asymmetry indices following the addition of unilateral complex training to regular basketball practice. One potential explanation for these adaptations may relate to neural factors associated with unilateral force production and the bilateral deficit phenomenon, whereby the force produced during simultaneous bilateral contractions can be lower than the summed force generated by each limb independently [30]. Unilateral exercises require each limb to generate force independently and may therefore provide a distinct neuromuscular stimulus. However, because neural activation, interhemispheric inhibition, and motor unit recruitment were not directly assessed in the present study, these mechanisms should be regarded as possible explanations rather than mechanisms demonstrated by our findings. The reductions observed in bilateral quadriceps and hamstring strength asymmetry should likewise be interpreted specifically in terms of the measured asymmetry indices. Although inter-limb asymmetry has been discussed in relation to athletic performance and injury-related outcomes, the clinical and performance relevance of a single fixed asymmetry threshold, such as 10%, is context-dependent and may vary according to the population, muscle group, and assessment method [17,18]. In the present study, mean bilateral quadriceps asymmetry in the experimental group decreased to 7.05% following the intervention; however, this finding should not be interpreted as evidence of reduced injury risk. Injury incidence and prospective injury surveillance were not included in the present study. Therefore, future prospective studies incorporating systematic injury surveillance are required to determine whether reductions in strength asymmetry are associated with subsequent changes in injury incidence [19].

The findings of the present study have several practical implications for strength and conditioning coaches, sport scientists, athletic trainers, and rehabilitation professionals. First, the unilateral complex training protocol consisted of two additional training sessions per week (45–55 min each) and was successfully integrated into the athletes’ regular basketball training schedule. This relatively time-efficient format may offer a feasible option for incorporating additional resistance and plyometric training within competitive basketball schedules [10]. However, given the absence of a time- and volume-matched active control condition, these practical implications should be interpreted as reflecting the addition of the complete training program to regular basketball practice rather than the isolated effects of its unilateral or complex components. Second, the present findings highlight the potential value of monitoring inter-limb strength asymmetry alongside traditional performance outcomes. Practitioners may consider incorporating limb-specific assessments, such as isokinetic dynamometry or appropriate single-leg field-based tests, into athlete monitoring to provide complementary information regarding side-to-side strength differences. Third, individualized unilateral loading may provide a practical means of applying different training volumes to each limb when limb-specific strength differences are identified. In the present intervention, the training volume was prescribed according to individually determined limb dominance; however, the current findings do not establish that preferential loading of a particular limb independently accounted for the observed adaptations. Finally, an improvement in the H/Q ratio was observed specifically for the left limb. Because the intervention comprised multiple resistance and plyometric exercises, the present design does not allow this finding to be attributed specifically to the posterior-chain exercises included in the program. Furthermore, injury incidence was not assessed; therefore, the observed changes in strength asymmetry and H/Q ratio should not be interpreted as evidence of injury-risk reduction. Overall, the present findings suggest that adding this unilateral complex training program to regular basketball practice may be a feasible approach for improving selected strength, asymmetry, and performance outcomes in competitive young male basketball players, although future studies using active, time- and volume-matched comparison groups are needed to determine the specific contribution of the training method.

Several limitations of the present study should be acknowledged when interpreting the findings. First, an important limitation of the present study is the absence of an active, time- and volume-matched control condition. The experimental group completed two additional unilateral complex training sessions per week alongside regular basketball practice, whereas the control group continued regular basketball training without any additional structured training stimulus. Consequently, the observed between-group differences should be interpreted as adaptations associated with adding the complete unilateral complex training program to regular basketball practice rather than as evidence of the specific effects of its unilateral or complex components. The additional training exposure itself may have contributed to the observed adaptations. Future randomized studies should therefore include an active comparator matched for training duration and volume, such as an alternative resistance, plyometric, or bilateral training program, to better isolate the specific contribution of unilateral complex training.

Second, the sample consisted exclusively of competitive young male basketball players competing in the Turkish Basketball Youth League (BGL) and U18 League. Therefore, the results cannot be directly generalized to female athletes, different age groups, professional players, or athletes from other sports.

Third, although the sample size was determined a priori using statistical power analysis, the relatively small number of participants may have limited the ability to detect small-to-moderate intervention effects, particularly for variables such as the right hamstring-to-quadriceps (H/Q) ratio and peak force. Consequently, replication with larger samples is warranted.

Fourth, the eight-week intervention may not have been sufficient to fully capture long-term neuromuscular and structural adaptations, such as muscle hypertrophy, tendon remodeling, or persistent changes in muscle architecture. In addition, no follow-up assessment was performed; therefore, the long-term retention of the observed improvements remains unknown. Habitual basketball training load was not quantitatively monitored using internal or external load measures; therefore, potential individual or between-group differences in background training exposure cannot be completely excluded. Finally, the physiological mechanisms proposed in this study remain speculative because they were not directly assessed. Variables such as electromyographic activity, rate of force development (RFD), ground reaction forces, three-dimensional biomechanical analysis, muscle architecture (e.g., fascicle length and pennation angle), tendon mechanical properties, and hormonal or metabolic responses were not evaluated. Future research incorporating these measurements would provide a more comprehensive understanding of the neuromuscular and structural adaptations induced by unilateral complex training. Furthermore, longitudinal investigations involving female athletes, different competitive levels, and extended follow-up periods are needed to determine the generalizability and long-term effectiveness of this training approach.

## 5. Conclusions

The present study showed that the addition of an eight-week unilateral complex training program to regular basketball practice was associated with improvements in selected lower-limb isokinetic strength outcomes, reductions in inter-limb strength asymmetry, improvement in the left hamstring-to-quadriceps (H/Q) ratio, and enhanced sprint and jump performance in competitive young male basketball players compared with regular basketball training alone. The H/Q response was side-specific, as a significant Group × Time interaction was observed for the left limb, whereas the corresponding interaction for the right limb was not significant. However, because the control group did not receive an additional time- and volume-matched training intervention, the observed between-group differences cannot be attributed specifically to the unilateral or complex characteristics of the program independently of the additional training exposure. From a practical perspective, the intervention consisted of two additional training sessions per week and was successfully integrated into the athletes’ regular basketball training schedule, indicating its practical feasibility within the training context examined. The observed reductions in inter-limb strength asymmetry indicate improvements in the measured asymmetry indices; however, whether such changes translate into a reduced risk of injury cannot be determined from the present study because injury incidence was not assessed. Future studies should incorporate active control conditions matched for training duration and volume to isolate the specific contribution of unilateral complex training. Further research incorporating electromyography, biomechanical analyses, muscle architecture assessments, injury surveillance, and long-term follow-up is also warranted to better characterize the mechanisms and potential practical implications of the observed adaptations. Replication in female athletes, athletes from different competitive levels, and other team sports is needed to determine the generalizability of these findings.

## Figures and Tables

**Table 1:** Exercise selection, training loads, progression, and weekly program structure.

**Monday Training (Workout A)**						
**Exercise**	**Set (*n*)**	**Rep/Time**	**Intensity (1RM/RPE)**			**Rest (s)**
	**Week 1–8**	**Week 1–8**	**Week 1–2**	**Week 3–6**	**Week 7–8**	
Bulgarian Split Squat	3 dominant/5 non-dominant	5/each side	80%	85%	85%	120
Rear Leg Elevated Split Jump	3 dominant/5 non-dominant	10/each side	BM—Maximum Effort			180
Unilateral Deadlift	3 dominant/5 non-dominant	5/each side	7 RPE	8 RPE	8 RPE	120
Single-arm Alternate Leg Bound	3 dominant/5 non-dominant	10/each side	BM—Maximum Effort			180
**Thursday Training (Workout B)**						
**Exercise**	**Set (*n*)**	**Rep/Time**	**Intensity (1RM/RPE)**			**Rest (s)**
	**Week 1–8**	**Week 1–8**	**Week 1–2**	**Week 3–6**	**Week 7–8**	
Single Leg Reserve Lunge	3 dominant/5 non-dominant	5/each side	7 RPE	8 RPE	8 RPE	120
Single Leg Vertical Jump	3 dominant/5 non-dominant	10/each side	BM—Maximum Effort			180
Unilateral Deadlift	3 dominant/5 non-dominant	5/each side	7 RPE	8 RPE	8 RPE	120
Single-arm Alternate Leg Bound	3 dominant/5 non-dominant	10/each side	BM—Maximum Effort			180

Rep = Repetitions; 1RM = One-repetition maximum; RPE = Rate of Perceived Exertion; BM = Body Mass.

**Table 2:** Baseline Characteristics and Outcome Measures of the Participants.

	Experimental Group (*n*:15) (X ± SD)	Control Group (*n*:14) (X ± SD)	*t*	*p*
**Age (Years)**	19.13 ± 0.99	19.14 ± 1.03	−0.025	0.980
**Height (cm)**	191.00 ± 7.14	188.14 ± 7.48	1.052	0.302
**Body Mass (kg)**	80.44 ± 7.87	79.11 ± 6.82	0.483	0.633
**BMI (kg·m^−2^)**	22.10 ± 2.00	22.20 ± 1.47	−0.154	0.879
**Right Ext (Nm)**	271.13 ± 35.18	267.57 ± 38.68	0.260	0.797
**Right Flex (Nm)**	153.20 ± 19.08	152.21 ± 14.72	0.155	0.878
**Left Ext (Nm)**	269.80 ± 35.64	272.79 ± 34.04	−0.230	0.820
**Left Flex (Nm)**	151.40 ± 20.57	155.50 ± 16.75	−0.586	0.563
**Bilateral Q (%)**	13.32 ± 2.55	12.54 ± 4.21	0.614	0.545
**Bilateral H (%)**	14.24 ± 3.80	13.23 ± 3.80	0.719	0.478
**H/Q—Right (%)**	57.02 ± 7.44	57.83 ± 9.04	−0.263	0.794
**H/Q—Left (%)**	56.46 ± 6.72	57.36 ± 5.47	−0.393	0.697
**CMJ (cm)**	36.13 ± 4.93	36.50 ± 4.91	0.894	0.379
**Peak Force (N)**	858.80 ± 76.45	854.14 ± 74.69	0.166	0.870
**Flight Time (ms)**	588.67 ± 37.77	563.57 ± 49.86	1.534	0.137
**Time to Takeoff (ms)**	802.67 ± 75.35	790.71 ± 96.51	0.373	0.712
**Abalakov Jump Test (cm)**	44.80 ± 5.75	43.79 ± 5.04	0.504	0.619
**20-m Sprint (s)**	3.03 ± 0.11	3.00 ± 0.08	0.745	0.463

Data are presented as mean ± SD. Between-group comparisons were performed using independent-samples *t*-tests. No statistically significant baseline differences were observed between groups (all *p* > 0.05).

**Table 3:** Two-Way Mixed-Design ANOVA Results for Isokinetic Strength Variables.

Variable	Group	Pre Test (X ± SD)	Post Test (X ± SD)	%Δ	Within Group d	d (EG-CG)		*p*-Value (ηp^2^) [95% CI]					
						Pre	Post	Time F(1,27), *p*	ηp^2^ [95% CI]	Group F(1,27), *p*	ηp^2^ [95% CI]	Interaction F(1,27), *p*	ηp^2^ [95% CI]
**Right Ext** **(Nm)**	EG (*n*:15)	271.13 ± 35.18	285.33 ± 29.75	5.24	0.67	0.10	0.44	6.029 **(0.021)**	0.18 [0.00–0.41]	0.510 (0.481)	0.02 [0.00–0.20]	2.157 (0.154)	0.07 [0.00–0.29]
	CG (*n*:14)	267.57 ± 38.68	271.14 ± 35.52	1.33	0.20								
**Right Flex (Nm)**	EG (*n*:15)	153.20 ± 19.08	168.73 ± 15.13	10.14	0.95	0.06	1.10	11.286 (**0.002**)	0.30 [0.04–0.51]	2.332 (0.138)	0.08 [0.00–0.30]	6.122 (**0.020**)	0.19 [0.00–0.41]
	CG (*n*:14)	152.21 ± 14.72	154.57 ± 9.91	1.55	0.20								
**Left Ext** **(Nm)**	EG (*n*:15)	269.80 ± 35.64	279.33 ± 32.87	3.53	0.76	0.09	0.24	2.497 (0.126)	0.09 [0.00–0.31]	0.047 (0.830)	0.01 [0.00–0.12]	6.038 (**0.021**)	0.18 [0.00–0.41]
	CG (*n*:14)	272.79 ± 34.04	270.71 ± 39.78	−0.76	0.16								
**Left Flex** **(Nm)**	EG (*n*:15)	151.40 ± 20.57	169.00 ± 17.64	11.62	1.90	0.22	0.82	21.318 (**0.001**)	0.44 [0.15–0.62]	0.683 (0.416)	0.03 [0.00–0.21]	31.513 (**0.001**)	0.54 [0.25–0.69]
	CG (*n*:14)	155.50 ± 16.75	153.79 ± 19.42	−1.10	0.19								

EG = Experimental Group; CG = Control Group; ηp^2^ = partial eta-squared; 95% CI = 95% Confidence Interval.

**Table 4:** Two-Way Mixed-Design ANOVA Results for Lower Extremity Asymmetry Variables.

Variable	Group	Pre Test (X ± SD)	Post Test (X ± SD)	%Δ	Within Group d	d (EG vs. CG)		*p*-Value (ηp^2^) [95% CI]					
						Pre	Post	Time F(1,27), *p*	ηp^2^ [95% CI]	Group F(1,27), *p*	ηp^2^ [95% CI]	Interaction F(1,27), *p*	ηp^2^ [95% CI]
**Bilateral Q (%)**	EG (*n*:15)	13.32 ± 2.55	7.05 ± 4.23	−47.07	1.31	0.23	1.76	6.363 (**0.018**)	0.19 [0.00–0.42]	9.882 (**0.004**)	0.29 [0.03–0.49]	15.580 (**0.001**)	0.37 [0.09–0.57]
	CG (*n*:14)	12.54 ± 4.21	13.92 ± 3.53	11.00	0.25								
**Bilateral H (%)**	EG (*n*:15)	14.24 ± 3.80	5.21 ± 4.62	−63.41	1.63	0.27	1.72	19.830 (**0.001**)	0.42 [0.14–0.61]	9.008 (**0.006**)	0.25 [0.02–0.47]	16.270 (**0.001**)	0.38 [0.10–0.57]
	CG (*n*:14)	13.23 ± 3.80	12.78 ± 4.13	−3.40	0.08								
**H/Q—Right (%)**	EG (*n*:15)	57.02 ± 7.44	59.33 ± 4.12	4.05	0.39	0.10	0.28	0.896 (0.352)	0.03 [0.00–0.23]	0.028 (0.869)	0.01 [0.00–0.10]	1.036 (0.318)	0.04 [0.00–0.24]
	CG (*n*:14)	57.83 ± 9.04	57.74 ± 7.10	−0.16	0.01								
**H/Q—Left (%)**	EG (*n*:15)	56.46 ± 6.72	60.74 ± 4.78	7.58	0.85	0.15	0.56	5.756 (**0.024**)	0.18 [0.00–0.40]	0.361 (0.553)	0.01 [0.00–0.18]	5.791 (**0.023**)	0.18 [0.00–0.41]
	CG (*n*:14)	57.36 ± 5.47	57.35 ± 7.10	−0.02	0.01								

EG = Experimental Group; CG = Control Group; ηp^2^ = partial eta-squared; 95% CI = 95% Confidence Interval.

**Table 5:** Two-Way Mixed-Design ANOVA Results for Physical Performance Variables.

Variable	G	Pre Test (X ± SD)	Post Test (X ± SD)	%Δ	Within Group d	d (EG vs. CG)		*p*-Value (ηp^2^) [95% CI]					
						Pre	Post	Time F(1,27), *p*	ηp^2^ [CI]	Group F(1,27), *p*	ηp^2^ [CI]	Interaction F(1,27), *p*	ηp^2^ [CI]
**CMJ (cm)**	EG	38.13 ± 4.93	41.07 ± 4.27	7.71	1.25	0.33	0.80	18.340 (**0.001**)	0.40 [0.12–0.59]	2.379 (0.135)	0.08 [0.00–0.30]	4.431 (**0.045**)	0.14 [0.00–0.37]
	CG	36.50 ± 4.91	37.50 ± 4.69	2.74	0.38								
**Peak Force (N)**	EG	858.80 ± 76.45	904.60 ± 95.05	5.33	0.80	0.06	0.44	10.612 (**0.003**)	0.28 [0.04–0.50]	0.530 (0.473)	0.02 [0.00–0.20]	2.919 (0.099)	0.10 [0.00–0.32]
	CG	854.14 ± 74.69	868.43 ± 67.83	1.67	0.36								
**Flight Time (ms)**	EG	588.67 ± 37.77	666.00 ± 67.06	13.13	1.98	0.57	1.23	49.295 (**0.001**)	0.65 [0.39–0.76]	7.576 (**0.010**)	0.22 [0.01–0.44]	9.587 (**0.005**)	0.26 [0.03–0.48]
	CG	563.57 ± 49.86	593.57 ± 48.30	5.32	0.69								
**Time to Takeoff (ms)**	EG	802.67 ± 75.35	712.00 ± 78.67	−11.30	1.24	0.14	1.01	7.162 (**0.013**)	0.21 [0.01–0.44]	1.827 (0.188)	0.06 [0.00–0.28]	12.277 (**0.002**)	0.31 [0.06–0.52]
	CG	790.71 ± 96.51	802.86 ± 100.03	1.54	0.14								
**Abalakov JT (cm)**	EG	44.80 ± 5.75	48.27 ± 5.54	7.75	2.66	0.19	0.58	48.744 (**0.001**)	0.64 [0.38–0.76]	1.100 (0.304)	0.04 [0.00–0.24]	10.866 (**0.003**)	0.28 [0.04–0.50]
	CG	43.79 ± 5.04	45.07 ± 5.54	2.92	0.57								
**20-m Sprint (s)**	EG	3.03 ± 0.11	2.96 ± 0.11	−2.31	0.61	0.28	0.62	1.712 (0.202)	0.06 [0.00–0.27]	0.280 (0.601)	0.01 [0.00–0.17]	5.511 (**0.026**)	0.17 [0.00–0.40]
	CG	3.02 ± 0.08	3.01 ± 0.08	0.33	0.22								

EG = Experimental Group; CG = Control Group; ηp^2^ = partial eta-squared; 95% CI = 95% Confidence Interval; ms = millisecond.

## Data Availability

The data presented in this study are available on request from the corresponding author. The data are not publicly available due to privacy restrictions.

## Supplementary Materials

The following supporting information can be downloaded at: https://www.mdpi.com/article/10.3390/life16091492/s1, Table S1: Bonferroni-Adjusted Simple-Effects/Pairwise Comparisons Following Significant Group × Time Interactions.
