# Supplementary material for: Effects of Eight Weeks of Unilateral Complex Training on Musculoskeletal Function, Inter-Limb Strength Asymmetry, and Athletic Performance in Competitive Basketball Players
Source: Life (Basel). 2026 Sep 6;16(9):1492. doi: 10.3390/life16091492 (PMC13608072; doi:10.3390/life16091492)
Supplement: Supplementary file 1 [file life-16-01492-s001.zip › life-4507951-supplementary.pdf]

Supplementary Table S1. Bonferroni-Adjusted Simple-Effects/Pairwise Comparisons Following Significant Group × Time Interactions

A. Isokinetic Strength Variables

Variable	Comparison	MD	SE	Adjusted p	95% CI for Mean Difference	
					Lower Bound	Upper Bound
Right Flex (Nm)	EG: Pre vs Post	-15.533	3.700	**0.001**	-23.125	-7.941
	CG: Pre vs Post	-2.357	3.830	0.543	-10.216	5.501
	Pre: EG vs CG	0.986	6.362	0.878	-12.069	14.040
	Post: EG vs CG	14.162	4.786	**0.006**	4.342	23.982
Left Ext (Nm)	EG: Pre vs Post	-9.533	3.281	**0.007**	-16.266	-2.801
	CG: Pre vs Post	2.071	3.396	0.547	-4.898	9.040
	Pre: EG vs CG	-2.986	12.961	0.820	-29.579	23.607
	Post: EG vs CG	8.619	13.407	0.526	-18.891	36.129
Left Flex (Nm)	EG: Pre vs Post	-17.600	2.391	**0.001**	-22.505	-12.695
	CG: Pre vs Post	1.714	2.474	0.494	-3.363	6.791
	Pre: EG vs CG	-4.100	6.996	0.563	-18.454	10.254
	Post: EG vs CG	15.214	6.883	**0.036**	1.092	29.337

Abbreviations: EG, experimental group; CG, control group; CI, confidence interval; Q, quadriceps; H, hamstring; H/Q, hamstring-to-quadriceps ratio; CMJ, countermovement jump. p values were adjusted using the Bonferroni correction.

B. Lower-Extremity Asymmetry Variables

Variable	Comparison	Mean Difference	Std. Error	Adjusted p	95% CI for Mean Difference	
					Lower Bound	Upper Bound
**Bilateral Q (%)**	EG: Pre vs Post	6.277	1.348	**0.001**	3.510	9.043
	CG: Pre vs Post	-1.382	1.395	0.331	-4.245	1.481
	Pre: EG vs CG	0.788	1.283	0.545	-1.846	3.421
	Post: EG vs CG	-6.871	1.452	**0.001**	-9.850	-3.892
**Bilateral H (%)**	EG: Pre vs Post	9.033	1.479	**0.001**	5.998	12.067
	CG: Pre vs Post	0.446	1.531	0.773	-2.695	3.588
	Pre: EG vs CG	1.015	1.410	0.478	-1.879	3.909
	Post: EG vs CG	-7.572	1.632	**0.001**	-10.920	-4.224
**H/Q – Left (%)**	EG: Pre vs Post	-4.284	1.239	**0.002**	-6.826	-1.742
	CG: Pre vs Post	0.007	1.282	0.996	-2.625	2.638

	Pre: EG vs CG	-0.898	2.286	0.697	-5.589	3.792
	Post: EG vs CG	3.392	2.233	0.140	-1.190	7.974

C. Physical Performance Variables

Variable	Comparison	Mean Dif.	SE	Adjusted p	95% CI for Mean Difference	
					Lower Bound	Upper Bound
**CMJ (cm)**	EG: Pre vs Post	-2.933	0.638	**0.001**	-4.243	-1.624
	CG: Pre vs Post	-1.000	0.661	0.142	-2.355	0.355
	Pre: EG vs CG	1.633	1.828	0.379	-2.117	5.384
	Post: EG vs CG	3.567	1.663	**0.041**	0.155	6.978
**Flight Time (ms)**	EG: Pre vs Post	-77.333	10.622	**0.001**	-99.128	-55.539
	CG: Pre vs Post	-30.000	10.995	**0.011**	-52.559	-7.441
	Pre: EG vs CG	25.095	16.354	0.137	-58.652	8.461
	Post: EG vs CG	72.429	21.843	**0.003**	27.611	117.246
**Time to Takeoff (ms)**	EG: Pre vs Post	90.667	20.387	**0.001**	48.836	132.497
	CG: Pre vs Post	-12.143	21.102	0.570	-55.442	31.156
	Pre: EG vs CG	11.952	32.030	0.712	-53.768	77.673
	Post: EG vs CG	-90.857	33.294	**0.011**	-159.170	-22.544
**Abalakov Jump Test (cm)**	EG: Pre vs Post	-3.467	0.473	**0.001**	-4.437	-2.496
	CG: Pre vs Post	-1.286	0.490	**0.014**	-2.290	-0.281
	Pre: EG vs CG	1.014	2.014	0.619	-3.117	5.146
	Post: EG vs CG	3.195	2.058	0.132	-1.027	7.417
**20-m Sprint (s)**	EG: Pre vs Post	0.065	0.025	**0.014**	0.014	0.116
	CG: Pre vs Post	-0.019	0.026	0.476	-0.071	0.034
	Pre: EG vs CG	0.026	0.035	0.463	-0.046	0.098
	Post: EG vs CG	-0.058	0.035	0.109	-0.130	0.014
